# Immune Evasion and Recognition of the Syphilis Spirochete in Blood and Skin of Secondary Syphilis Patients: Two Immunologically Distinct Compartments

**DOI:** 10.1371/journal.pntd.0001717

**Published:** 2012-07-17

**Authors:** Adriana R. Cruz, Lady G. Ramirez, Ana V. Zuluaga, Allan Pillay, Christine Abreu, Carlos A. Valencia, Carson La Vake, Jorge L. Cervantes, Star Dunham-Ems, Richard Cartun, Domenico Mavilio, Justin D. Radolf, Juan C. Salazar

**Affiliations:** 1 Centro Internacional de Entrenamiento e Investigaciones Médicas (CIDEIM), Cali, Colombia; 2 Centers for Disease Control and Prevention, Atlanta, Georgia, United States of America; 3 Clinical Research Center, University of Connecticut Health Center, Farmington, Connecticut, United States of America; 4 Department of Pediatrics, University of Connecticut Health Center, Farmington, Connecticut, United States of America; 5 Department of Medicine, University of Connecticut Health Center, Farmington, Connecticut, United States of America; 6 Department of Pathology, Hartford Hospital, Hartford, Connecticut, United States of America; 7 Unit of Clinical and Experimental Immunology, Humanitas Clinical and Research Center, Milan, Italy; 8 Department of Immunology, University of Connecticut Health Center, Farmington, Connecticut, United States of America; 9 Department of Genetics and Developmental Biology, University of Connecticut Health Center, Farmington, Connecticut, United States of America; 10 Division of Pediatric Infectious Diseases, Connecticut Children's Medical Center, Hartford, Connecticut, United States of America; University of Washington, United States of America

## Abstract

**Background:**

The clinical syndrome associated with secondary syphilis (SS) reflects the propensity of *Treponema pallidum* (*Tp*) to escape immune recognition while simultaneously inducing inflammation.

**Methods:**

To better understand the duality of immune evasion and immune recognition in human syphilis, herein we used a combination of flow cytometry, immunohistochemistry (IHC), and transcriptional profiling to study the immune response in the blood and skin of 27 HIV(-) SS patients in relation to spirochetal burdens. *Ex vivo* opsonophagocytosis assays using human syphilitic sera (HSS) were performed to model spirochete-monocyte/macrophage interactions *in vivo*.

**Results:**

Despite the presence of low-level spirochetemia, as well as immunophenotypic changes suggestive of monocyte activation, we did not detect systemic cytokine production. SS subjects had substantial decreases in circulating DCs and in IFNγ-producing and cytotoxic NK-cells, along with an emergent CD56−/CD16+ NK-cell subset in blood. Skin lesions, which had visible *Tp* by IHC and substantial amounts of *Tp*-DNA, had large numbers of macrophages (CD68+), a relative increase in CD8+ T-cells over CD4+ T-cells and were enriched for CD56+ NK-cells. Skin lesions contained transcripts for cytokines (IFN-γ, TNF-α), chemokines (CCL2, CXCL10), macrophage and DC activation markers (CD40, CD86), Fc-mediated phagocytosis receptors (FcγRI, FcγR3), IFN-β and effector molecules associated with CD8 and NK-cell cytotoxic responses. While HSS promoted uptake of *Tp* in conjunction with monocyte activation, most spirochetes were not internalized.

**Conclusions:**

Our findings support the importance of macrophage driven opsonophagocytosis and cell mediated immunity in treponemal clearance, while suggesting that the balance between phagocytic uptake and evasion is influenced by the relative burdens of bacteria in blood and skin and the presence of *Tp* subpopulations with differential capacities for binding opsonic antibodies. They also bring to light the extent of the systemic innate and adaptive immunologic abnormalities that define the secondary stage of the disease, which in the skin of patients trends towards a T-cell cytolytic response.

## Author Summary

Syphilis, a sexually transmitted disease caused by the spirochetal bacterium *Treponema pallidum*, affects close to 10 million people per year worldwide. Despite the robust nature of the humoral and cellular immune responses associated with the disease, weeks to months may elapse before the host gains control of the infection. Moreover, in the absence of antibiotic treatment, containment is often incomplete and relapses are common. Herein we studied aspects of the immune response in the blood and skin of patients with secondary syphilis to better understand the factors that determine whether the bacterium evades host defenses or is cleared in its natural human host. Our findings support the importance of the macrophage as a primary means of bacterial killing in the skin, while suggesting that the extent of bacterial clearance is determined by the bacterial loads present in either the blood or skin of patients and the appearance of spirochetes which are resistant to uptake (phagocytosis) by the macrophages. Study results underscore the extent of the systemic immunologic abnormalities triggered by the bacterium and provide new insights regarding the complexity of the immune response in the skin of untreated patients.

## Introduction

Syphilis is a sexually transmitted multi-stage disease caused by the spirochetal bacterium *Treponema pallidum* (*Tp*), subspecies *pallidum* [1], [2]. Despite the existence of inexpensive and effective antibiotic treatment regimens, more than 10.5 million new syphilis cases are estimated to occur yearly throughout the world [3]. Infection begins when the bacterium comes into contact with skin or mucous membranes, multiplying locally, while simultaneously disseminating through blood vessels and lymphatics [1], [2], [4]–[6]. The distinctive painless ulcer (chancre) of primary syphilis typically appears 2–4 weeks after the initial contact with the spirochete [2], [6], [7]. By this time, organisms that have disseminated from the primary site of infection have invaded various organ tissues, most notably the skin [2], [6], setting the stage for what is classically known as secondary syphilis (SS). SS, the principal focus of the current study, characteristically presents with a variety of muco-cutaneous manifestations as well as systemic signs and symptoms within 4–10 weeks of the initial infection [4], [5], [8]. Despite the robust nature of the cellular and humoral immune responses associated with SS, weeks to months may elapse before lesions resolve. Infectious relapses are common during the first few years of infection [2], while approximately one-third of untreated patients develop one of the potentially devastating forms of recrudescent disease collectively referred as tertiary syphilis [9]. The factors that influence the complex and shifting balance between this persistent bacterium and host clearance mechanisms are not well understood.

*T. pallidum* contains abundant lipoproteins which are capable of activating macrophages and DCs via CD14 [10]–[13] and Toll-like receptor 1 (TLR1) and TLR2-dependent signaling pathways [11], [12], [14]–[16]; consequently, these pathogen associated molecular patterns (PAMPs) are believed to be major pro-inflammatory agonists during spirochetal infection [17]. However, due to the bacterium's unique outer membrane (OM) structure, which includes a lack of surface exposed lipoproteins [18]–[22], these PAMPs are not readily accessible to TLRs or other pattern recognition receptors (PRRs) present on monocytes/macrophages or dendritic cells (DCs). As a result, it is believed that spirochetes can replicate in tissues and disseminate without triggering innate pathogen recognition systems. Presumably, as local spirochetal burdens increase, a small number of organisms are taken up by tissue-based DCs; which then traffic to draining lymph nodes to present cognate treponemal antigens to naïve T and B-cells. The emergence of opsonic antibodies would then enhance uptake and degradation of the bacterium in tissues, allowing spirochetal PAMPs to gain access to PRRs lining the phagocytic vacuole and triggering their activation [23]. Because of the bacterium's extraordinarily low density of integral outer membrane proteins (OMPs) [1], [19], [24], [25] and the limited antibody responses they elicit in humans [24]–[26], anti-treponemal antibodies alone are unlikely to be sufficient to control bacterial replication and prevent further dissemination. In support of this idea, *ex vivo* opsonophagocytosis assays using either rabbit peritoneal macrophages [27] or human PBMCs [28] point out that even in the presence of syphilis immune sera, substantial numbers of spirochetes avoid phagocytosis. Lastly, findings from a recent study provide additional evidence that organisms within *Tp* populations differ widely with respect to the density of surface antigens recognized by syphilitic sera [25].

*T. pallidum* is capable of provoking an intense cellular immune response generally believed to be the cause of the tissue damage that gives rise to clinical manifestations [5]. The extent to which the diverse cellular components of syphilitic infiltrates contribute to clearance of spirochetes, however, remains an open question. In the rabbit model, the appearance of *Tp* reactive lymphocytes correlates with the progression of mononuclear cell infiltration and macrophage activation at the sites of experimental inoculation [29]–[31]. Immunohistochemistry (IHC) and RT-PCR analysis of biopsy specimens obtained from patients with primary and secondary syphilis lesions demonstrate that syphilitic skin lesions are also composed of lymphocytes and macrophages capable of expressing mRNA for the Th1 cytokines, IL-2, IFNγ and IL-12 [32], [33]. While helper T-cells outnumber cytolytic T-cells in experimentally infected rabbit tissues [34] and in human primary syphilitic lesions [35], equal or greater numbers of CD8+ T-cells characterize human SS syphilis inflammatory infiltrates [35]–[38]. The finding by Van Voorhis *et al* [32] that both perforin and granzyme B are expressed in human syphilis lesions supports the idea that in *Tp*-infected SS skin tissues cytolytic T-cells have a role in bacterial clearance. How CD8+ T-cells are activated in the skin is unclear given that this lymphocyte subset usually responds to antigens presented via the class I Major Histocompatibility Complex (MHC) pathway [39], which is generally not associated with control of extracellular pathogens like *Tp*.

Efforts to understand the duality of immune evasion and immune recognition in syphilis have been hindered by the inability to propagate the bacterium *in vitro* and the lack of a suitable inbred animal model for performing immunologic studies. To circumvent these problems and obtain information directly relevant to the disease process in humans, we have been studying SS, the stage in which the dichotomous features of syphilitic infection are clearly evident and specimens are readily obtainable. Herein, we used a combination of flow cytometry, IHC and transcriptional profiling to investigate key aspects of the innate and adaptive immune response in the blood and skin of untreated SS patients in relation to the spirochetal burdens present in each of these two immunologically distinct compartments. We then used our previously described *ex vivo* opsonophagocytosis assay [28], [40] to model spirochete-monocyte/macrophage interactions in the blood and skin. As a whole, our findings support the importance of opsonophagocytosis as a primary means for clearance of treponemes, while suggesting that the balance between phagocytic uptake and evasion is determined by the relative burdens of bacteria and the presence of *Tp* subpopulations with differential capacities for binding opsonic antibodies. The findings in the skin demonstrate that in addition to CD4+ and CD8+ T-cells, CD56+ NK-cells are also enriched and are thus likely to participate in activation of dermal macrophages through their ability to secrete IFN-γ. Unexpectedly, we discovered that patients have profound immunophenotypic alterations in circulating monocytes, DCs and NK-cells, including the emergence of a CD56^negative^CD16^high^ NK-cell subset that is known to be highly dysfunctional in patients with uncontrolled chronic viral infections [41], [42]. These findings reveal the extent of the systemic innate and adaptive immunologic abnormalities that define the secondary stage of the disease, which in the skin of patients trends towards a T-cell cytolytic response.

## Materials and Methods

### Human Subjects

Adult SS patients were identified and referred for enrollment through a previously described network of health care professionals in Cali, Colombia [8]. The diagnosis of SS was based on the medical history and compatible skin or mucosal lesions, reactive non-treponemal test (RPR, Rapid Plasma Reagin titer ≥1∶8) and a positive confirmatory treponemal test (FTA-ABS, Fluorescent Treponemal Antibody Test Absorbed). All serological tests were performed at a reference laboratory in Colombia (Clínica Colsanitas). Patients were excluded if they were known to be HIV-positive, if they had serologic evidence of current or prior infection with hepatitis B or hepatitis C, were receiving anti-inflammatory or immunosuppressive medications, had recently used antibiotics, or had a history of chronic dermatitis or other underlying acute or chronic disease. Peripheral whole blood samples obtained from enrolled patients, along with 4-mm punch skin biopsies from secondary syphilis lesions from a subset of these patients, were processed for immunological and molecular assays as described below. All patients were treated with 2.4 million units of intramuscular benzathine penicillin as recommended by Colombian public health standards, which are in accord with available CDC treatment guidelines. Patients were asked to return two months after receiving antibiotic treatment for a clinical and immunological follow-up. Healthy control volunteers (non-reactive RPR, negative FTA-ABS, non-HIV/HBV/HCV), of similar background and socio-economic status, were recruited by the study site in Cali. Healthy volunteers, with no serologic evidence of prior or current syphilis, were recruited at the University of Connecticut Health Center (UCHC) to serve as controls for the *ex vivo Tp*-monocyte stimulation experiments (described below). The Institutional Review Boards of, Centro Internacional de Entrenamiento e Investigaciones Médicas (CIDEIM) in Cali, Colombia, the Connecticut Children's Medical Center (CCMC), UCHC and the Center for Diseases Control and Prevention (CDC) approved all relevant study protocols. All healthy volunteer and syphilis patients, regardless of whether they were enrolled by the Cali site or at UCHC, gave voluntary written informed consent to participate in the study.

A total of 27 HIV-negative SS patients were eligible for participation. Clinical and epidemiologic features for these patients are summarized in **Table 1**. Peripheral blood mononuclear cells (PBMCs) obtained from whole blood samples from these patients were examined by flow cytometry and RT-PCR at the time of enrollment as described below. Flow cytometric analysis was repeated in a subset (n = 13) of enrolled SS patients approximately 60 days after receiving antibiotic treatment. A total of 12 of the 27 SS patients also had skin biopsies processed for targeted array analysis (12/12); skin biopsies were also studied by IHC in four of the twelve patients. We previously reported quantitative *Tp* DNA results from whole blood samples obtained from all 27 SS patients studied herein [8]. In the current study we also determined spirochetal burdens in 4 SS skin lesion samples from these same patients and that also were studied by IHC. A total of 26 healthy volunteers were enrolled at the Cali site; 23 controls were included for flow cytometric immunologic studies and three additional subjects provided healthy skin control samples for microarray analysis (see below).

**Table 1:** Clinical and laboratory characteristics of secondary syphilis patients.

	(n = 27)
**Gender**	
Male	8 (30%)
Female	19 (70%)
**Age**	
Age, mean (range), years	36(19–64)
**Race**	
White	0
Mestizo	20 (74%)
Black	7 (26%)
**Clinical Findings**	
Duration of skin rash, mean (range), days	40 (7–120)
Skin lesions	
Plaques on palms and/or soles	19 (70%)
Moth eaten alopecia	1 (4%)
Codylomata lata	5 (19%)
Mucosal lesions	8 (30%)
Mild flu-like symptoms (i.e. headache, myalgias)	20 (74%)
Adenopathy	13 (48%)
**Laboratory Findings**	
RPR titer	
>1∶64	21 (78%)
>1∶16 but <1∶64	5 (19%)
>1∶8 but <1∶16	1 (4%)
Erythrosedimentation rate (ESR) >15	18 (67%)
Lymphopenia (<30%)	12 (44%)
Anemia (Hgb <12)	8 (30%)
***polA*** **+PCR** *	
Peripheral Blood	
Positive total samples studied (n = 25)	12/25 (44%)
Positive when DNA extracted fresh (n = 11)	7/11 (66%)
Tp *polA* copies: monocyte ratio (“MOI”)	0.3–0.9∶1
Secondary syphilis skin lesions	
Positive (n = 12)	8/12 (67%)

*: Diagnostic PCR results previously published in PLoS NTD (Ref 8).

### Propagation and Harvesting of *T. pallidum*

Live *Tp* (Nichols strain) was used for the monocyte simulation experiments on the same day of the extraction from rabbit testicles as previously described [28]. All animal experimentation was conducted following the NIH guidelines for housing and care of laboratory animals and was performed in accordance with the UCHC institutional regulations after review and approval by Institutional Animal Care and Use Committee.

### Monocyte Isolation and Stimulation

Highly purified human monocytes were isolated from healthy volunteer PBMCs using a magnetic cell sorting monocyte isolation kit (Miltenyi Biotech, Auburnas) as previously described [17]. Cells were plated and incubated with 10% heat inactivated normal human sera (NHS) or human syphilitic sera (HSS) for 8-hours at 37°C/5% CO_2_ with fresh *Tp* at multiplicities of infection (MOIs) of 1, 10 and 30. In some assays, 100 ng/ml of LPS (Sigma-Aldrich) was used as a positive control for cytokine production. At the end of the 8-hr incubation period, cells were harvested for flow cytometry, epifluorescence and confocal microscopy. Supernatants were collected for cytokine analysis and *Tp* counting. Experiments with HSS were performed using a pool of sera from a group of HIV-seronegative SS patients as previously reported [28]. All culture media and reagents utilized in the stimulation experiments were confirmed to be free of LPS contamination (<10 pg/ml) by Limulus amoebocyte lysate assay quantification (Cambrex, MA).

### Cell staining and Flow Cytometry

Isolated monocytes from healthy US volunteers and PBMCs from SS syphilis patients and controls, where processed for flow cytometry as previously described [43]. The antibody panels used in for flow cytometry are listed in **Table 2**. Surface staining procedures were done as previously described [28]. Individual cell populations were selectively gated for analysis based on the expression of corresponding immuno-phenotypes. Multiparameter files were analyzed using WINMDI v2.8 software (Joseph Trotter, Scripps Clinic).

**Table 2:** Antibody staining panels used for flow cytometric analysis.

*Experiment*	*Panel*	*Fluorochrome Label*				*Immunophenotypes*
		FITC	Phycoerythrin	PerCP	APC	
Isolated monocytes (*ex vivo* experiments)	1	CD40	CD83		CD14	Activated monocytes
PBMCs (SS patients)	1	CD14	D83 isotype		CD40 isotype	Isotype control
	2	CD14	CD83		CD40	Activated monocytes
	3	Lineage cocktail	CD83 isotype	HLA-DR	CD11c	Isotype control
	4	Lineage cocktail	CD83	HLA-DR	CD11c	Activated dendritic cells (DCs)
	5	CD56	CD16		CD3	NK-cells

### Opsonization Assays

Human PBMCs obtained from healthy US volunteers were plated and stimulated with freshly extracted *Tp* Nichols strain at 37°C/5% CO_2_. Selected samples were incubated with 10% heat inactivated (56°C for 30 min) NHS or with 10% heat inactivated HSS obtained from individual SS patients or pooled samples from Cali SS patients. Samples were incubated in the presence of LysoTracker Red endosomal dye (Molecular Probes), and harvested after a 4-hr incubation period. *Tp*–cell associations were visualized by immunofluorescence assay (IFA) as previously described [28]. Images were acquired on an Olympus BX41 epifluorescence microscope equipped with a Retiga Exi CCD camera (QImaging) and processed with ImageJ 1.40 (NIH, USA). To quantitate spirochetal uptake, up to 10 fields were selected sequentially and monocytes containing internalized and degraded spirochetes in the form of fluorescent blebs were counted using images acquired by epifluorescence microscopy. A total of 100 cells were counted for isolated monocyte experiments. After an 8-hr incubation period 10 µl aliquots from *Tp*-stimulated-monocyte supernatants were enumerated, in triplicate, by dark-field microscopy on a Petroff-Hausser counting chamber. Percentage of bacterial recovery was calculated using a “time zero” spirochetal count.

### Serum and Ex-Vivo Stimulation Supernatant Cytokine Analysis

Simultaneous measurements of TNF-α, IL1-β, IL-6 and IL-10 were performed in supernatants from *ex vivo* experiments and in individual SS patient's serum, using a Human Inflammatory Cytokine Bead Array (CBA) per the manufacturer's (BD) protocol.

### Quantitative Real Time Reverse Transcriptase PCR (qRT-PCR)

Isolated PBMCs (2×10^6^ cells) from SS patients and healthy controls were stored in 300 µl of RNA later at −80°C until processing. RNA was extracted at the Cali site using the RNeasy Mini Kit (Qiagen) according to the manufacturer's protocol. Up- or down-regulation of selected transcripts were measured in Complementary DNA (cDNA) from *ex vivo Tp*-monocyte stimulation experiments for selected genes by quantitative RT-PCR (qRT-PCR) analyses. RNA was extracted from both stimulated and unstimulated cells using the Paxgene blood RNA kit (Qiagen, Valencia, CA). The quality of the RNA was verified both with the DU 530 Life Science spectrophotometer (Beckman, Fullerton, CA) and Agillent Bioanalyzer. cDNA was prepared from both patient and healthy donor extracted RNA samples using a high capacity cDNA RT kit. (Qiagen, Foster City, CA). Commercially available gene expression assays (Applied Biosystems) were used for amplification of the following transcripts; TNF-α (Hs00174128_m1), IL-1β (Hs00174097_m1), IL-6 (Hs00985639_m1), IL10 (Hs00174086_m1Hs), IFN-β (Hs00277188_s1), TLR2 (Hs00610101_m1), TLR7 (Hs00152971_m1), TLR8 (Hs00152972_m1), TLR9 (Hs00152973_m1), CD40 (Hs00374176_m1), IL-17 (Hs99999082_m1) and IFN-γ (Hs00174143_m1). qRT-PCR gene expression assays for the house keeping gene, glyceraldehyde-3-phosphate dehydrogenase (GAPDH) (Hs99999905_m1), were performed using identical aliquots of each cDNA as normalization controls. All amplification reactions were performed in triplicate; control reactions without reverse transcriptase also were performed to confirm the absence of contaminating DNA. Expression levels of all transcripts studied were normalized to the GAPDH level and the relative changes in gene expression were calculated using the 2^−ΔΔCt^ method [44].

### DNA Extraction from SS Skin Biopsies

DNA from tissues (15–25 mg) was extracted using the QIAamp DNA minikit (QIAGEN Inc., Valencia, CA) following procedures recommended by the manufacturer. DNA was eluted from the QIAGEN columns in 100 µl of elution buffer at 70°C and stored at −80°C. The concentration of DNA was determined spectrophotometrically by the 260/280 nm absorbance. The quality and integrity of the DNA were determined by electrophoretic fractionation of 5 µl of extracted DNA through 1.2% agarose gels (E-gels: Invitrogen Corp., Carlsbad, CA) at 70 V for 30 min.

### Measurement of Spirochetal Burdens by Quantitative Real-Time PCR

PCR amplification of the *Tp polA* gene was performed using forward primer TP-1 (5′CAGGATCCGGCATATGTCC3′), reverse primer TP-2 (5′AAGTGTGAGCGTCTCATCATTCC3′), and probe TP-3 (5′CTGTCATGCACCA GCTTCGACGTCTT3′) as previously published [45], with some exceptions. The probe was labeled with Cyanine (Cy5) at the 5′ end and black-hole quencher 3 (BHQ3) at the 3′ end. Thermocycling was performed in a Rotor-Gene 6000 instrument (Qiagen, Valencia, CA) as follows: two hold cycles at 50°C for 2 min and 95°C for 10 min, respectively; and 45 cycles of 95°C for 15 sec and 60°C for 1 min. Each PCR run included positive and negative (no template) control reactions. The *Tp* copy numbers for each skin biopsy specimen were extrapolated from the standard curve generated using ten-fold serial dilutions of purified *Tp* DNA. The raw data obtained from the amplifications were adjusted for quantity tested to generate the *polA* DNA concentration, expressed as copies/ml or copies/µg of extracted cellular DNA from tissues.

### Skin Biopsy Arrays

A 4-mm punch biopsy from SS skin lesions was obtained from a group of 12 patients and from normal skin from 3 healthy Colombian controls (see above), snap-frozen and stored in liquid nitrogen in preparation for overnight transportation on dry ice from Cali to UCHC. Upon arrival at UCHC, tissues were homogenized in Trizol (Invitrogen), RNA was isolated, cleaned with Turbo DNase (Ambion, Applied Biosystems) followed by cDNA synthesis using High Capacity cDNA Archive Kit (Applied Biosystems) according to the manufactures instructions. Gene transcripts were amplified per manufacturer's instructions (Applied Biosystems) using two commercially available array kits; TaqMan® Human Immune Array and TaqMan® Human Phagocytosis Array. Briefly, the array was performed in a 2 µL reaction volume containing 62.5 pg of cDNA, 1 µL of water and 1 uL of gene expression master mix; and the Phagocytosis Array was processed in a 20 µL reaction volume containing 5 ng of cDNA, 10 µL water and 10 µL Gene Expression Master Mix. Amplification reactions were performed with 7900HT Fast Real Time (Applied Biosystems) using the following conditions: 95°C for 20 min, and 40 cycles of 95°C for 1 s and 60°C for 20 sec. Expression levels of all transcripts studied were normalized to the GAPDH level and the relative changes in gene expression generated between 12 SS patients and three healthy controls were calculated with the 2^−ΔΔCt^ method using DataAssist™ v2.0 Software (Applied Biosystems). Up- or down-regulation of gene transcripts for this analysis were considered significant if their expression pattern in tissue was at least 2-fold higher or lower than control skin-samples and if the p-value was <0.05. Selected gene transcripts were confirmed by conventional RT-PCR as described above.

### Immunohistochemistry (IHC)

Paraffin embedded skin biopsies which were available from 4/12 patients studied by microarray (see above), were immunohistochemically labeled with antibodies against CD4, CD8, CD56, CD11c, CD14 and CD68 using an automated IHC staining platform (Bond Max, Leica-Microsystems, Buffalo Grove, IL). IHC staining for *Tp* was manually performed at room temperature. Slides sections were depparaffinized in xylene (Allegiance Healthcare Corporation, McGaw Park, IL) and rehydrated in graded alcohol to water. After quenching endogenous peroxidase activity and a wash in phosphate-buffered saline (PBS), the slides were incubated for 30 minutes at room temperature with a rabbit polyclonal anti-*Tp* antibody (dilution 1∶500; Biocare, Concord, CA). Following a short wash in PBS, slides were covered with anti-rabbit EnVision+ detection system (Dako, Carpinteria, CA) for 30 minutes. Following a final wash in PBS, slides were incubated with the AEC+ (aminoethylcarbazole) chromogen (Dako) for 10 minutes, rinsed and counterstained in hematoxylin. Positive and negative controls were run in parallel for each of the antibodies used. For evaluation of immunofluorescence results biopsy specimens were read in a blinded fashion by at least one independent investigator. Labeled cells were enumerated per visual field and expressed as a percentage of inflammatory cells per 200 cells counted per high power field (HPF).

### Statistical Analysis

Immunologic markers of interest were first compared between a group of healthy controls affiliated by employment to the CIDEIM facility and healthy controls of similar socio-economic background to SS patients enrolled in the study. Student *t* test or the equivalent non-parametric methods (i.e. Mann-Whitney *U*) test results allowed us to conclude that immunologic parameters of interest between the two healthy control populations were not statistically different (data not shown). Results from the combined control group were thus used for all comparisons between healthy volunteers and SS patients. Flow cytometry cell surface expression patterns of immunologic markers of interest and cytokine outputs were compared between patients and controls by using unpaired Student *t* tests or where indicated the equivalent non-parametric test (i.e. Wilcoxon). A two-tail statistical analysis was performed for all comparisons, except to analyze dose-responses in the *Tp*-monocyte stimulation assay results. For each analysis, both the standard deviation and the standard error of the mean (SEM) were calculated and *p* values of <0.05 were considered significant. Statistical analysis was done using GraphPad prism 4.0 (GraphPad Software, San Diego, CA).

## Results

### Immunophenotypic Alterations in Peripheral Blood Monocyte (PBMC) and dendritic Cells (DCs) in Untreated SS Patients

**Figure 1:**
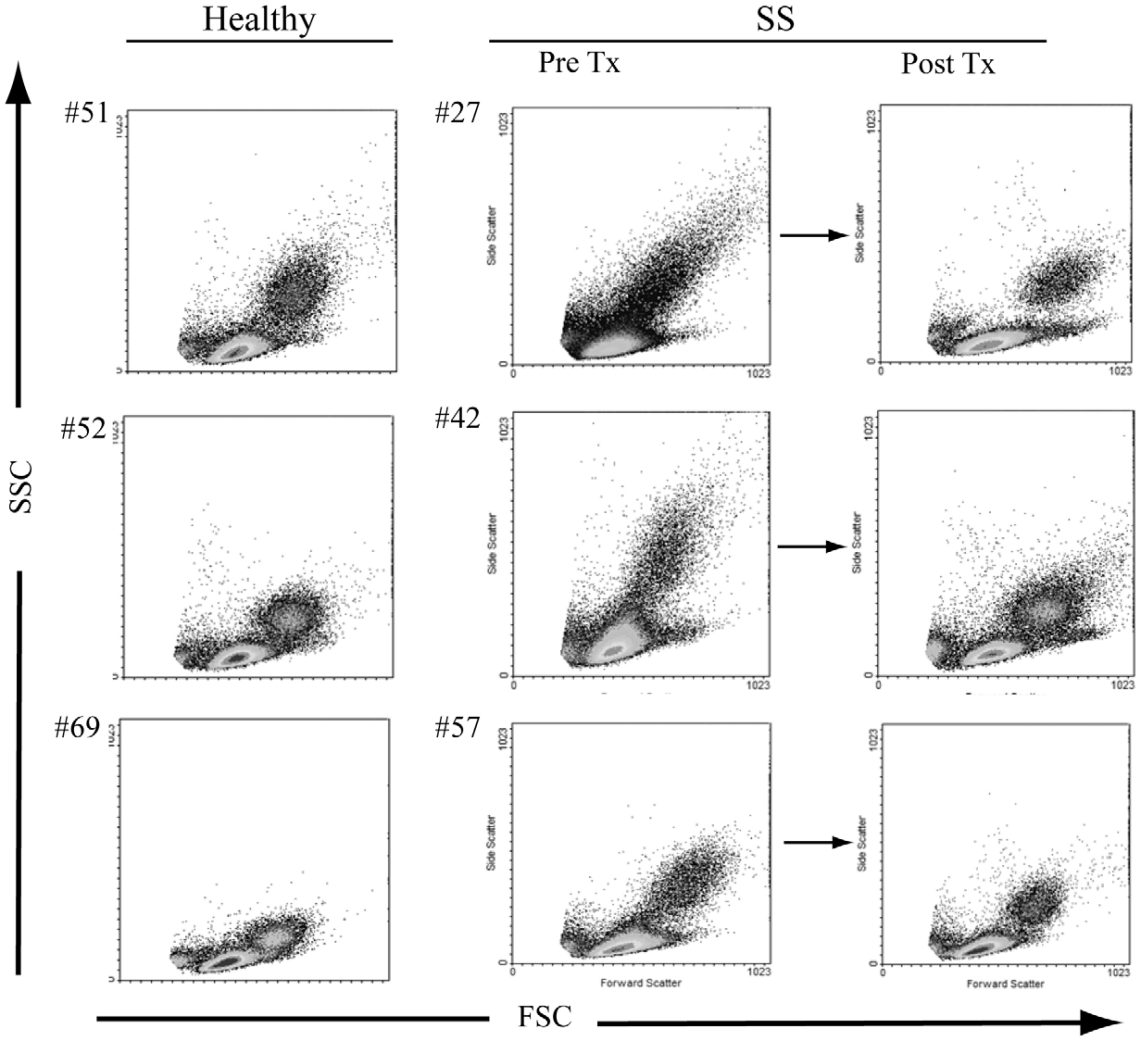
Secondary syphilis induces immunophenotypic alterations in human monocytes. Peripheral blood mononuclear cells (PBMCs) were isolated from secondary syphilis (SS) patients before and around 60 days after penicillin treatment and compared to healthy controls. The flow cytometry dot-plot reveals forward and side scatter features of gated monocytes from three different and representative patient samples. The dot plots reveal a population of monocytes with increased size and granularity in SS patients, which normalized at the follow-up visit.

**Figure 2:**
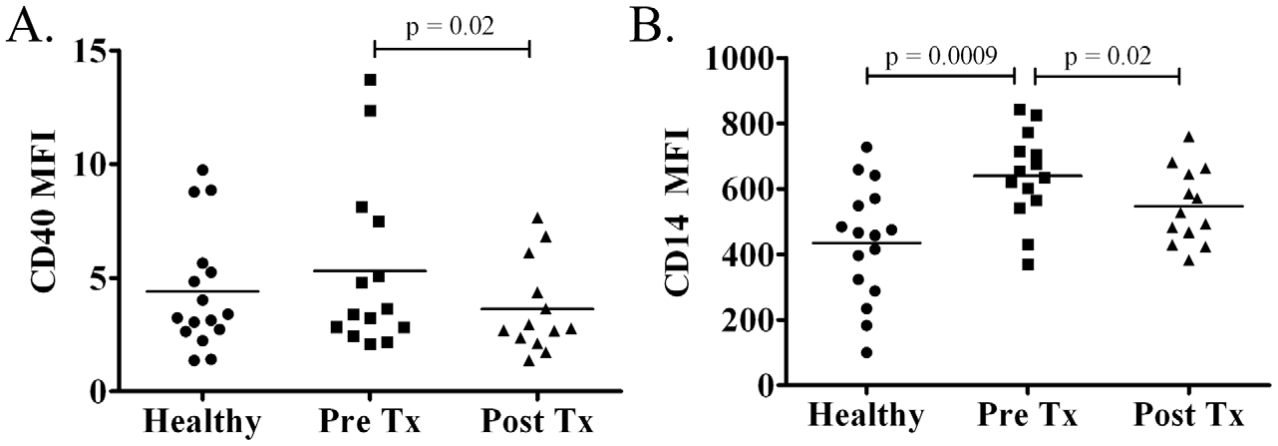
Cell surface activation markers in secondary syphilis patient monocytes. Cell surface activation markers were examined in monocytes from secondary syphilis (SS) patients before (Pre-Tx) and around 60 days after penicillin treatment (Post-Tx) and compared to healthy controls. (**A**) A modest but significant decrease in CD40 MFI was evident between paired acute and convalescent samples obtained from SS patients before and after treatment. (**B**) Significant increases in CD14 MFI expression were observed between syphilis patients and healthy volunteers prior to antibiotic treatment and between pre- and post-penicillin treatment (*p* values are shown in the figure).

**Figure 3:**
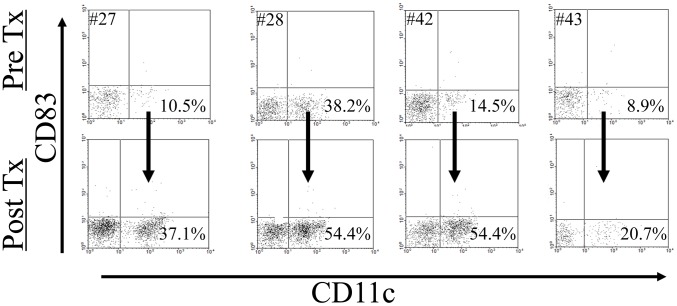
Immunophenotypic alterations in dendritic cell (DC) populations. Circulating DCs were analyzed by flow cytometry in secondary syphilis (SS) patients before (Pre-Tx) and after penicillin treatment (Post-Tx). DCs were characterized by flow cytometry parameters as being HLA-DR+ and Lineage cocktail negative (not shown) and the expression of CD11c into monocytoid (CD11c+) and plasmacytoid (CD11c−) and expression of the co-stimulatory molecule CD83. A marked decrease was observed in the CD11c+ population in the blood of 7/12 SS patients and at the follow-up visit this population recovered in these same SS patients.

We recently reported that a significant proportion of a cohort of untreated SS patients had low-level spirochetemia based on whole blood *Tp*-DNA quantitation [8]. This finding, together with our earlier demonstration that HSS induces opsonophagocytosis-dependent activation of monocytes and DCs in PBMCs [28], prompted us to examine whether circulating monocytes and DCs obtained from these same SS patients exhibited evidence of immune activation at the time of initial presentation. Unlike healthy volunteers, at study entry more than half (7/13) of the patients studied exhibited increases in the size and granularity in the total monocyte population, which were no longer present two months after penicillin treatment (**Figure 1**). Expression of the activation marker CD40 and mean fluorescence average values for CD14 (**Figure 2**) also were increased in monocytes from untreated SS patients; statistically significant post-treatment reductions confirmed that this finding was disease-specific. Despite these immunophenotypic alterations, there were no significant pre-treatment increases in selected cytokines (TNF, IL-1β, IL-10 or IL-6) based on either qRT-PCR analysis of isolated PBMCs or CBA of pre-treatment sera (data not shown). We also studied circulating DCs, using expression of CD11c [46], [47] to distinguish monocytoid (CD11c+) and plasmacytoid (CD11c−) DCs. As depicted in the representative flow cytometry dot plots in **Figure 3**, 67% (10/15) of the patients studied exhibited a selective decrease in the proportion of CD11c+ DCs (<35%), which corrected in all but three patients at the follow-up visit. Neither DC population displayed increased expression of the activation marker CD83 (**Figure 3**).

### Emergence of a CD56^negative^ NK-cell Population in SS Patients

**Figure 4:**
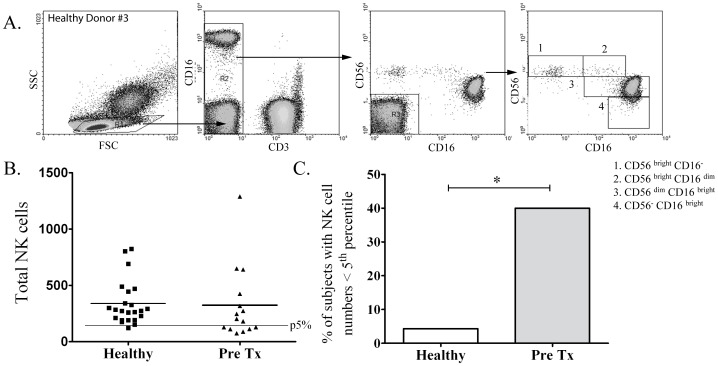
Secondary syphilis (SS) patients exhibit a significant decrease in total NK-cell populations. (**A**) Gating procedure to determine NK-cells subsets according to CD56 and CD16 expression by flow cytometry are shown. (**B and C**) A significantly larger percentage of SS subjects exhibit NK-cell values below the 5^th^ percentile of established published normal values (line depicts the cutoff), when compared to healthy controls (* indicates *p*<0.05).

**Figure 5:**
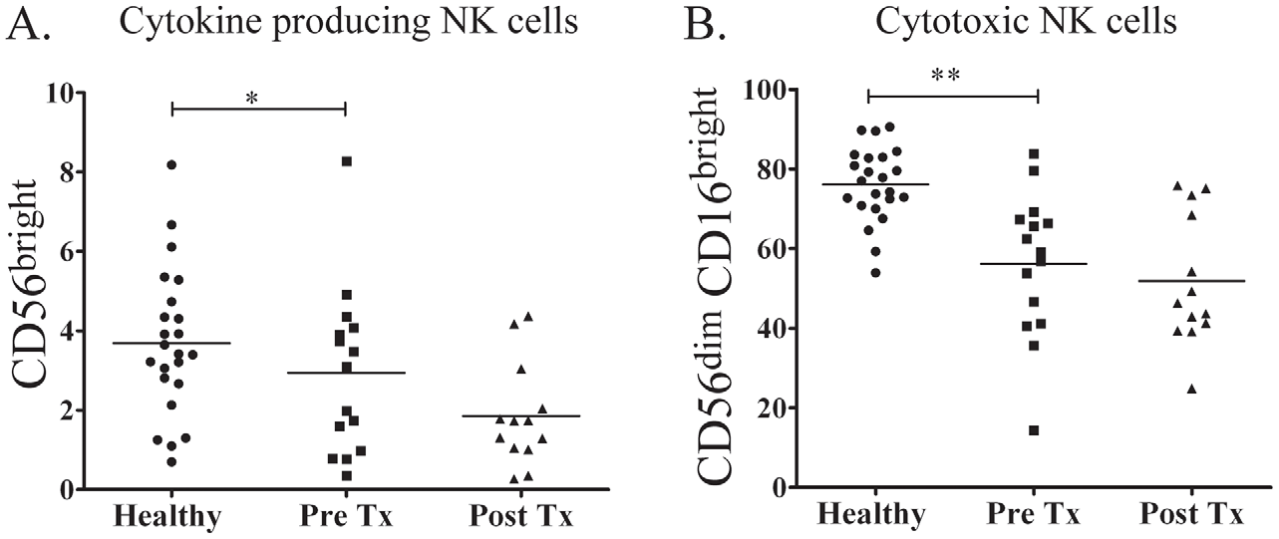
NK-cell subsets distribution in secondary syphilis (SS) patients. SS patients exhibit significant decreases in (**A**) cytokine-producing NK-cells (* *p* = 0.02) and (**B**) cytotoxic NK-cells (* *p*<0.001). Values are shown at enrollment (Pre-Tx) and after penicillin treatment (Post-Tx). Neither cell subset appears to recover following treatment.

**Figure 6:**
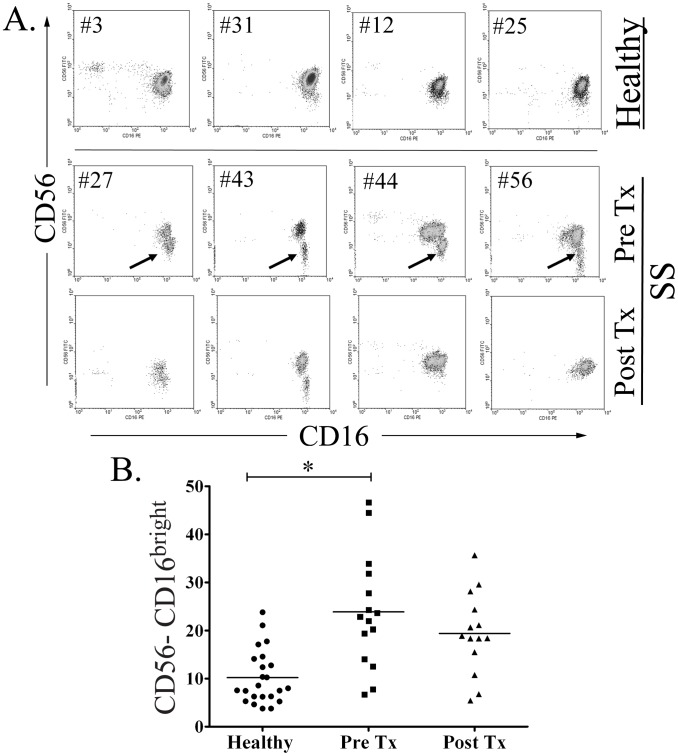
Emergence of a CD56-negative NK-cell population in secondary syphilis (SS) patients. (**A**) The CD56^negative^ CD16+ NK-cells subset in untreated SS patients are shown by the black arrows. (**B**) Significant increases (* *p* = 0.003) in CD56^ negative^ CD16+ NK-cell population was seen in SS patients. This anomaly was not present in healthy controls.

NK-cells play a critical role in the immune response to human pathogens by secreting IFN-γ and other immunomodulatory molecules [48], [49] and by promoting T-cell polarization and DC maturation [50]. The finding that NK-cells are the principal source of IFN-γ in *Tp*-stimulated PBMCs [28], together with existing evidence that total NK-cell numbers and function may be altered during SS [51], [52], prompted us to study circulating NK-cell subsets in the blood of our patients. NK-cells were classified by flow cytometry using a previously described scheme according to their relative expression of CD16 and CD56, (**Figure 4A**) [53], [54]. CD56^bright^ cells are known to be potent cytokine producers with limited cytotoxic activity, while CD56^dim^ cells have strong cytotoxic capacity but a decreased ability to produce cytokines [53], [54]. As displayed in **Figure 4B–C**, when compared to healthy controls, a significantly greater percentage of SS patients had total circulating NK-cell values below the 5^th^ percentile of published normal adult NK-cell numbers [55] (40% vs. 4.3% respectively, p = 0.01). Significant decreases in IFN-γ-producing (CD56^bright^) and cytotoxic (CD56^dim^CD16^bright^) NK-cell subsets were largely responsible for the decline in total NK-cell values (**Figure 5**). By contrast the CD56^negative^CD16^bright^ NK-cell subset, a recently described NK-cell population which exhibits both poor cytolytic activity and impaired cytokine production [41], was significantly increased in most untreated SS patients (**Figure 6**) but returned to near normal values in all patients at the follow-up visit.

### Substantial Numbers of Spirochetes Co-Exist within a Mixed Cellular Infiltrate in Skin Lesions from SS Patients

**Figure 7:**
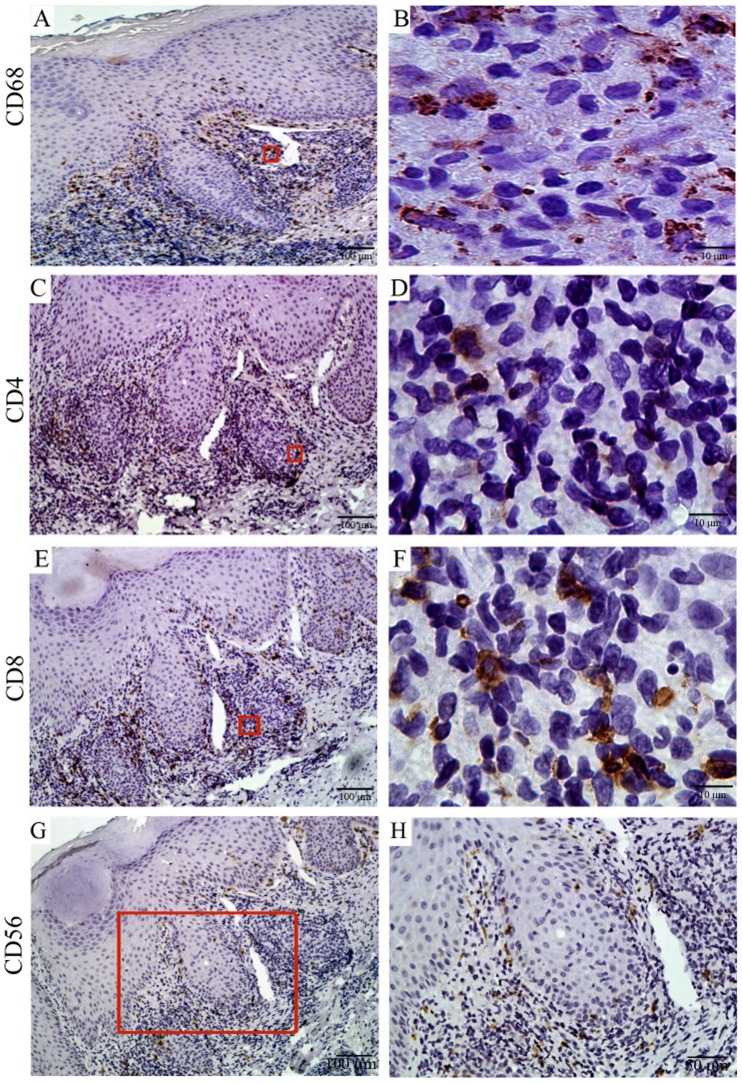
Immunophenotypic cellular composition of the inflammatory infiltrate in secondary syphilis patient skin lesions. IHC staining depicts CD68+ macrophages (**A** and **B**), CD4+ T-cells (**C** and **D**), CD8+ T-cells (**E** and **F**) and CD56+ NK-cells (**G** and **H**). Panels B, D, E and H are high magnification images of the red boxed areas in A, C, E and G.

**Figure 8:**
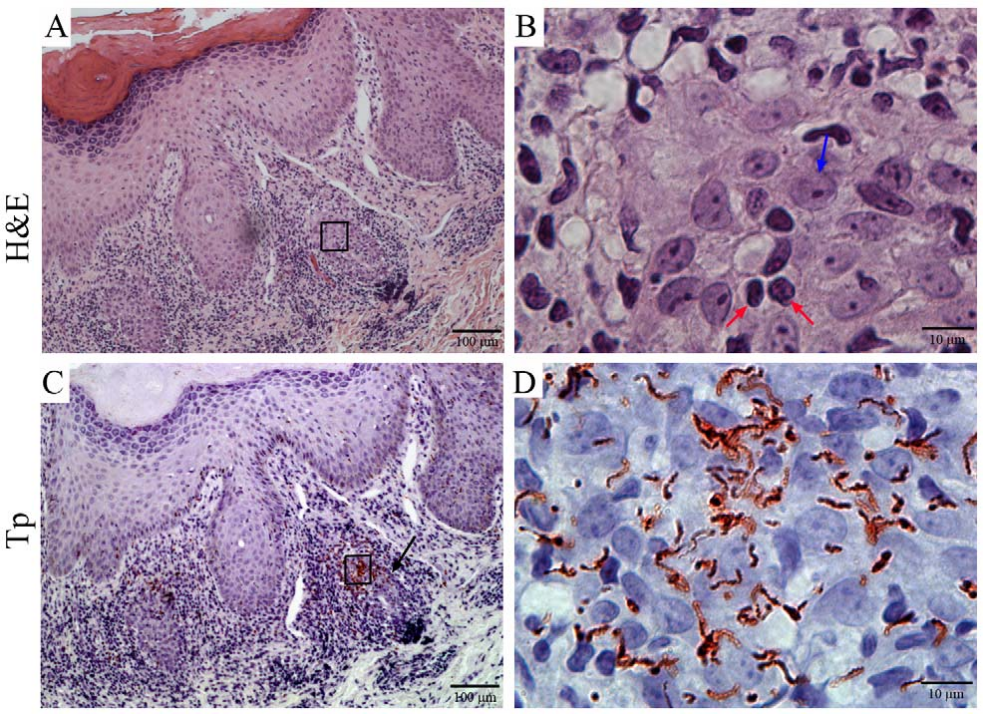
Spirochetal clusters are present in secondary syphilis skin lesions. Representative skin biopsy from a posterior neck secondary syphilis (SS) lesion was processed for IHC. (**A/B**) H&E stain of SS lesions. (**C/D**) IHC staining reveals abundant spirochetes embedded within a mixed cellular inflammatory infiltrate (shown in the red box) in the papillary dermis. The blue arrow points to a tissue histiocyte and the read arrows to two dermal lymphocytes.

We previously reported [8] that the routine histology for the patients described in this study was characteristic of typical SS lesions [2], [5], [32], [33], [56], [57]. Herein, we used IHC staining techniques to analyze four SS skin biopsies and corroborate that the cellular infiltrates were in agreement with previously published IHC analysis [32], [38], [56], [58] and to explore potential mechanisms for immune recognition of spirochetes within tissues. Substantial numbers of dermal mononuclear cells expressed the macrophage marker CD68 (Table S3 and **Figure 7A and B**) [59]. Staining with two other macrophage markers (CD11c and CD14) revealed very similar patterns (data not shown). Syphilitic lesions were also comprised of CD4+ and CD8+ lymphocytes (**Figure 7B–E**), with the CD8+ phenotype predominating in three of the four biopsies [36] (**Table S1**). Interestingly, 5% of dermal mononuclear cells expressed the NK-cell marker CD56^+^, an approximate five-fold increase from normal percentages (<1%) of NK-cell values in healthy skin [48] (**Figure 7F and G** **and Table S1**). Because of the lack of specific markers for CD56^negative^CD16^high^ NK-cells, we were unable to determine if this unexpected circulating NK-cell phenotype was also present in the skin of SS patients. Several recent studies have also called attention to the sensitivity of IHC for detection of spirochetes in tissues in addition to its well-recognized ability to provide information regarding the spatial relations between *Tp* and cellular infiltrates in the skin [2], [5], [32], [33], [57]. In agreement with these prior reports, dense clusters of spirochetes could be seen in a perivascular location within the papillary dermis in close physical proximity to aggregates of lymphocytes and histiocytes (**Figure 8**). Spirochetes were also visualized in the mid- and deep-layers of the dermis away from infiltrating cells, straddling the dermal-epidermal interface, and within the lower layers of the epidermis (data not shown). Lastly, we confirmed that all four biopsies studied had *Tp* DNA by quantitative PCR analyses (**Table S1**).

### Microarray Analysis of Cutaneous Lesions

Transcriptional analysis of SS skin biopsies has thus far been limited to a small number of gene products [32], [38]. In this study, we used transcriptional profiling to gain additional insights into the molecular mechanisms underlying the inflammatory responses elicited by spirochetes in skin. **Table 3** highlights key transcripts associated with the array, while the complete list is presented in **Tables S2 and S3**. Consistent with the finding by IHC that syphilitic lesions contain an abundance of macrophages, the transcript for CD68 [59] was significantly up-regulated. Also upregulated were transcripts for the macrophage activation markers CD40 [60], CD80 and CD86 [61], a number of cytokines known to be secreted by human monocytes/macrophages in response to opsonized *Tp* (TNFα, IL-6, IL-1β and IL-10), and numerous other molecules associated with macrophage activation. Interestingly, transcripts for both TLR1 and TLR2, which are required for recognition of treponemal lipoproteins by monocytes/macrophages [28], were up-regulated, whereas TLR6, which recognizes diacylated lipoproteins in association with TLR1 [62], was not. Transcripts for three different FCγ phagocytic receptors, FCγR1A/C (CD64), FCγR2A (CD32), and FCγR3A/B (CD16) also were significantly over-expressed in lesional skin.

**Table 3:** Transcriptional Profile - Secondary syphilis skin biopsies (n = 12) vs healthy control skin (n = 3).

Gene Transcript	Name	Fold Increase	p value
***PATHOGEN RECOGNITION***			
***Macrophage and Dendritic Cell Activation***			
CD40	CD40	2	<0.05
CD80	B7-1	129	<0.05
CD86	B7-2	6	<0.05
Fc γ Receptors			
FcGR1A/FcGR1C	CD64	84	<0.01
FcGR2A	CD32	7	<0.01
FcGR3A/FcGR3B	CD16	21	<0.01
***Toll-like receptors***			
TLR1	Toll-Like-Receptor 1	6	<0.01
TLR2	Toll-Like-Receptor 2	2	<0.05
TLR7	Toll-Like-Receptor 7	19	<0.01
TLR8	Toll-Like-Receptor 8	22	<0.01
TLR9	Toll-Like-Receptor 9	19	<0.01
***Scavenger Receptors***			
CD68	Scavenger Receptor Class D	12	<0.01
***CYTOKINES and GROWTH FACTORS***			
TNF	Tumor Necrosis Factor	9	<0.01
IL6	Interleukin 6	9	<0.01
IL1B	Interleukin 1β	17	<0.01
IL10	Interleukin 10	27	<0.01
IFNG	Interferon γ	6	<0.05
IL-15	Interleukin 15	13	<0.05
IL17	Interleukin 17	2	<0.05
IL2RA	CD25, Interleukin 2 receptor A	8	<0.05
IL4	Interleukin 4	35	>0.05
TGFB1	Transforming Growth Factor	3	<0.01
***CHEMOKINES***			
CCL2	MCP-1, Macrophage inflammatory protein-1	7	<0.01
CCL3	MIP-1a, Macrophage inflammatory protein-1α	115	<0.05
CCL5	RANTES, Regulated upon Activation, Normal T-cell Expressed, and Secreted	63	<0.01
CCL19	MIP-3b, Macrophage inflammatory protein-3b, SLC	8	<0.05
CXCL10	IP-10, IFNγ induced protein 10kDa, CRG-2	387	<0.01
CXCL11	IP-9, IFNγ induced protein 9, I-TAC	226	<0.01
CCR4	C-C chemokine receptor type 4, CD194	3	<0.05
CCR5	C-C chemokine receptor type 5, CD195	10	<0.01
CCR7	C-C chemokine receptor type 7, CD197	18	<0.01
***LYMPHOCYTES***			
CD3E	CD3	13	<0.01
CD8A	CD8	177	<0.01
CD4	CD4	7	<0.01
CD19	CD19	27	<0.05
CD28	CD28	9	<0.01
CD38	CD38	52	<0.01
***CYTOLYTIC ACTIVITY***			
GNLY	Granulysin	85	<0.01
GZMB	Granzyme B	78	<0.01
PRF1	Perforin	18	<0.01

Transcripts for the T-cell receptors CD3, CD4 and CD8, as well as the T-cell activation marker CD38, were all markedly increased in lesional biopsies (**Table 3**). IFNγ, a potent macrophage activator that can be produced by CD4+ and CD8+ memory T-cells as well as NK-cells, also was significantly up-regulated in lesional biopsies. The transcript for IL-17 also was expressed in the lesional biopsies, which is in agreement with a recent report that IL-17+ T-cells are present in the skin of SS patients [38]. Of particular interest, we saw a dramatic increase in expression of transcripts for granulysin, perforin and granzyme B, which can be produced by both NK-cells [63] and CD8+ T-cells [64], thus, providing evidence for a strong cytotoxic response.

Type I IFNs modulate multiple aspects of innate and adaptive immunity in response to bacterial infections [23], [65], [66], including activation of NK-cells, DCs and macrophages. The arrays revealed marked increases in the expression of three endosomal TLRs, TLR-7, TLR-8 and TLR-9 [67], all of which are associated with the production of type I IFNs [65], [68]. TLR7 and TLR9 are expressed predominantly by plasmacytoid DCs, a subset which we previously have shown is enriched in SS skin lesions [69], whereas TLR8 is expressed by activated human macrophages [70]. Two type I IFN-inducible chemokines, CXCL10 (IP-10) and CXCL11 (IP-9), were significantly over-expressed in lesional biopsies. In line with these results, we found by RT-PCR that the transcript for IFN-β was also up-regulated in lesional biopsies (data not shown).

### Human Syphilitic Serum (HSS) Promotes Opsonophagocytosis of Treponemes and Monocyte Activation

**Figure 9:**
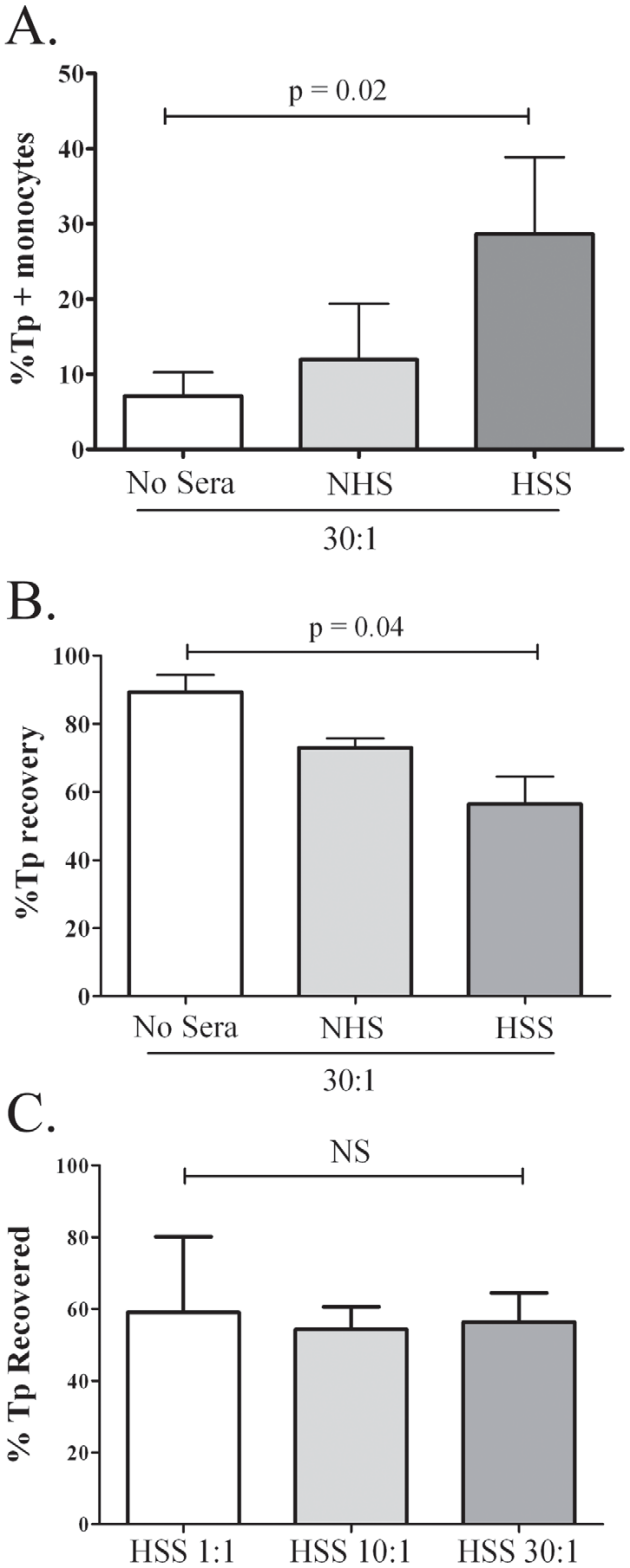
*T. pallidum* (*Tp*) uptake and recovery by IFA. Purified human monocytes obtained from healthy controls were stimulated with fresh *Tp* Nichols strain (MOI 1∶1, 10∶1 and 30∶1) were incubated for 8-hours alone or where indicated in the presence of 10% heat inactivated normal human serum (NHS) or human syphilitic serum (HSS). (**A**) Percentage of phagocytosed *Tp* was greater when HSS was present. (**B**) Percentage of non-phagocytosed *Tp* which were recovered in supernatants at the end of incubation time is shown in each of the two graphs. Spirochetal recovery was substantially higher in the absence of HSS; nonetheless more than half of the bacteria avoided recognition and uptake despite the presence of HSS (*p* values shown correspond to statistical comparisons between groups by ANOVA). (**C**) Percentage of spirochetes recovered was similar in the presence of HSS at three different MOIs.

**Figure 10:**
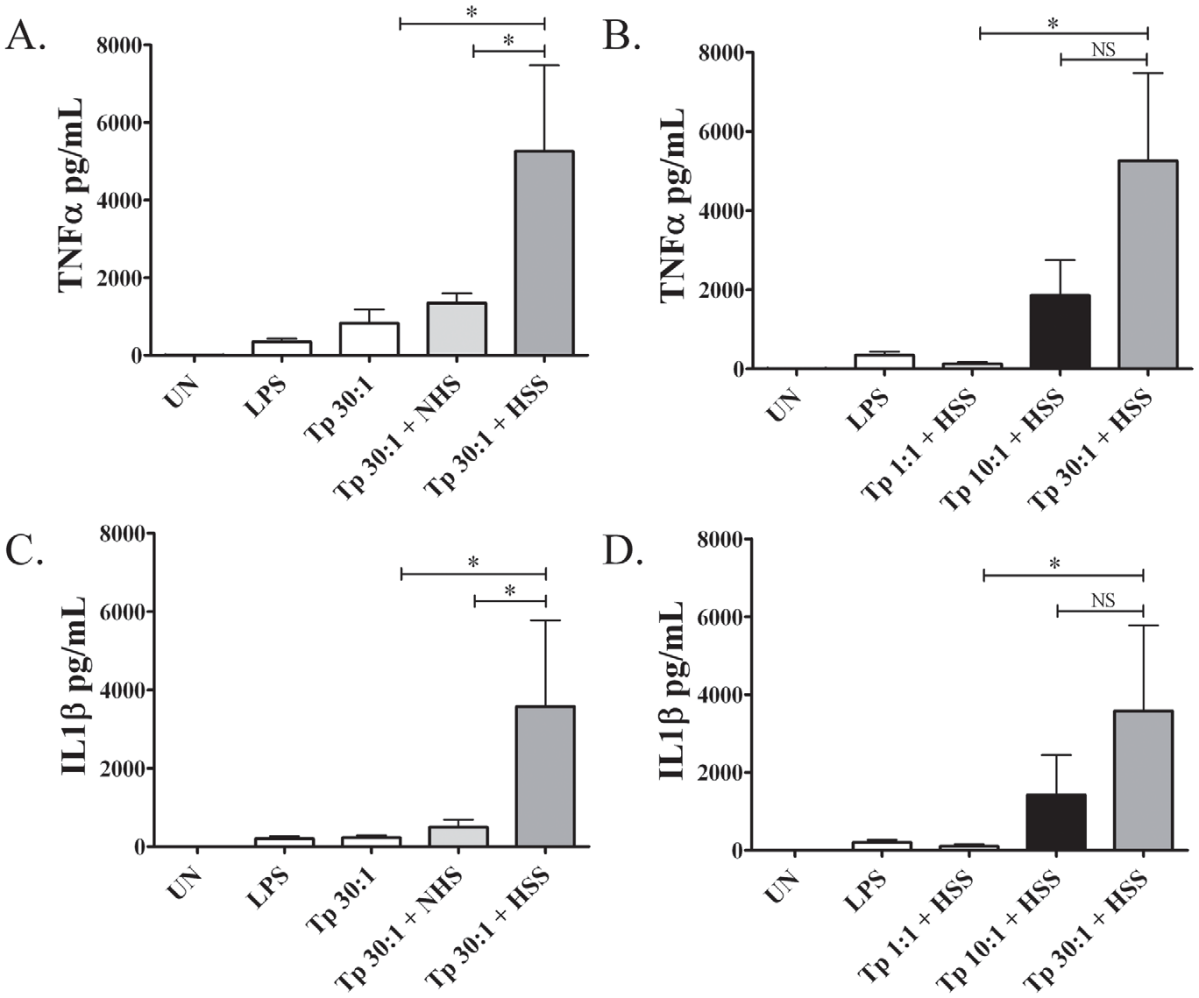
Cytokine output in response to opsonized *Tp*. Isolated human monocytes were stimulated for 8-hours with live *Tp* at three different spirochete to monocyte ratios (MOIs) (1∶1, 10∶1 and 30∶1) in the presence or absence of 10% heat inactivated human syphilitic serum (HSS). LPS was used as a positive control. Cytokines in supernatants were quantitated in picograms (*pg*)/ml by cytokine bead array as described in Methods. **A, C**: Higher cytokine production (TNFα and IL-1β) was elicited when spirochete-stimulated monocytes were immersed in 10% heat inactivated HSS. **B, D**: Cytokine output was greater with higher MOIs (* indicates where *p* values are <0.05 between different conditions studied by paired or unpaired student's T-test analysis as described in the Methods, NS = non-significant differences).

Syphilitic antibodies are believed to play an essential role in both cellular activation and bacterial clearance by promoting opsonophagocytosis of the syphilis spirochete by macrophages [40], [57], [71], [72]. Herein, we used an *ex vivo* stimulation assay to model the effect of opsonic antibodies on spirochete-monocyte/macrophage interactions at graded MOIs as it may occur in the skin and blood of patients. In comparison to normal human sera, pooled syphilitic sera significantly enhanced monocyte uptake of *Tp* in a dose-dependent manner (**Figure 9A**). Similar results were obtained for serum specimens of 12 different patients confirming that opsonic antibodies are commonly produced in early syphilis (data not shown). Interestingly, even in the presence of HSS a large proportion (56%) of the spirochetes was not phagocytosed (**Figure 9B**). The observation that the percentage of spirochetes recovered was not significantly different between the MOIs of 1, 10 and 30 (**Figure 9C**), argues that the lack of uptake at the higher MOI is not due to FC-receptor saturation. As shown in **Figure 10**, opsonized *Tp* also induced a marked dose-dependent increase in secretion of TNF and IL-1β. Importantly, cytokine production was minimal at the lowest MOI (1∶1) tested, which is similar to the MOI in spirochetemic SS patients (**Table 1**). A similar dose-dependent increase was seen in the production of Il-6 and IL-10 and expression of the activation markers CD40 and CD83 (data not shown).

## Discussion

Venereal syphilis can be considered a contest between the ability of *T. pallidum* to avoid immune recognition and the adeptness of the host's innate and adaptive immune responses to “track down” and eliminate the spirochetal pathogen. To begin to understand the mechanisms that underlie the dichotomy between immune evasion and immune recognition of the syphilis bacterium, herein we compared key aspects of the innate and adaptive immune response in the blood and skin of SS patients, to spirochetal burdens present in these two immunologically distinct compartments. The evidence suggests that spirochetes circulate through the blood mostly unimpeded by host's immune defenses, while the larger burden of treponemes present in the skin elicit a highly complex inflammatory cellular immune response that paradoxically does not rapidly control spirochetal replication. Our results reinforce the importance of the macrophage in the immune response to *Tp* and establish that the balance between phagocytic uptake of the spirochete and its ability to evade innate immune recognition is influenced by the number of bacteria present in either the blood or the skin, as well as the emergence of *Tp*-subpopulations with differential capacities for binding opsonic antibodies. We hypothesize that the striking immunophenotypic alterations found in circulating innate immune cells in SS patients are not the result of their direct interaction with spirochetes in the blood, but instead a manifestation of the systemic effects of the bacterium in other tissues including the bone marrow. Lastly, we demonstrate that in addition to CD4+ and CD8+ T-cells, CD56+ NK-cells are enriched in *Tp*-infected skin lesions and, thus, could contribute to macrophage activation and bacterial clearance through their ability to secrete IFN-γ.

One of the most significant findings in this study is the extent of the immunophenotypic alterations that distinguish monocytes, DCs and NK-cells in the blood of untreated SS patients from those of healthy controls. Despite the demonstration that circulating monocytes were noticeably larger by flow cytometry and expressed higher levels of CD14 and CD40 than monocytes obtained from healthy volunteers, SS patients did not have measurable increases in transcription or secretion of monocyte-derived cytokines in their blood. The *ex vivo* finding that bacterial uptake and cytokine production was minimal at low spirochete-monocyte ratios (1∶1), an MOI that closely mirrors the calculated MOI in the blood of SS patients, may explain why low levels of spirochetes in the blood are unable to induce cytokine production by circulating monocytes. Given that similar morphologic changes were not evident *ex vivo Tp*-stimulated monocytes at a similar spirochete to cell ratio of 1∶1 (data not shown), leads us to conclude that the immunophenotypic alterations in circulating monocytes could be provoked directly by the spirochete in the blood. Instead they raise the possibility that the syphilis spirochete could be affecting macrophage-DC progenitor cells in the bone marrow, before they differentiate into CD14+ monocytes and mobilize into the blood stream [73]. The striking decrease in circulating CD11c+ DCs suggests that these cells are marshaled from the blood into infected skin tissues [74]. Our prior findings that monocytoid DCs obtained from the blood and skin of SS patients express high levels of the C-type lectin DC-SIGN [43], an adhesion molecule which is known to regulate DC trafficking from blood into tissues [75], supports the notion that this DC subset migrates into infected skin. A particularly novel finding in this study was the marked decrease in total circulating NK-cell numbers and the distinct emergence of a highly atypical circulating CD56^negative^CD16^bright^ NK-cell population. Mavilio and colleagues [41], [42] previously reported similar increases in HIV-infected patients with uncontrolled viremia and confirmed that this subset of NK-cells was not only poorly cytolytic but also had an impaired capacity to produce IFN-γ and other cytokines. Our report is the first to show that this abnormal NK-cell subset can also be increased in the course of a bacterial infection.

The critical role of the macrophage in the pathogenesis of venereal syphilis was initially ascertained from histological analysis of *Tp*-infected rabbit tissues [76], [77] and the finding that rabbit immune sera markedly enhanced spirochetal uptake and clearance by peritoneal macrophages *in vitro* [71]. Results from prior human studies are generally in line with those in the rabbit model in that they confirm that large numbers of macrophages and T-cells are also present in early syphilis lesions [36], [37], [56], [78]–[80]. Herein, we used a combination of IHC and transcriptional profiling to corroborate that macrophages are indeed the predominant inflammatory cell in the skin. The confirmation that HSS enhances uptake of spirochetes by isolated monocytes *ex vivo*, inducing their activation in a dose-dependent manner, underscores the importance of opsonophagocytosis in spirochetal recognition and clearance. While low-level spirochetemia seemingly facilitates chronic spread of the bacterium, in *Tp*-rich skin infiltrates opsonized spirochetes are more likely to be taken up by IFN-γ activated tissue macrophages. Paradoxically, the *ex vivo* model results also indicate that even at high MOIs, a large subset of the spirochetes avoid phagocytosis by monocytes. This finding is in accord with the observation in the rabbit model by Lukehart and co-workers [27] that a subpopulation of opsonic antibody-resistant spirochetes emerges during active infection. The same group has proposed that antigenic variation in candidate OMP antigenic targets (i.e. TprK) helps us understant how *Tp* evades host antibody responses [81]. Our own data suggests that an additional explanation is that *Tp* populations differ widely with respect to the density of surface antigens recognized by syphilitic serum [25], which would then allow populations of spirochetes to escape opsonization and avoid clearance.

The role of the adaptive cellular immune response in treponemal clearance has been studied in the rabbit model and in humans with active disease [2]. Replication of treponemes at the site of inoculation in rabbit tissues elicits an intense inflammatory response that histologically resembles a classic delayed type hypersensitivity reaction (DTH) [56], which in addition to macrophages is composed predominantly of CD4+ lymphocytes [34]. IHC and molecular studies in humans confirm that primary and SS lesions are also enriched for Th1-cytokine producing CD4+ lymphocytes [32], [33]. In contrast to the rabbit, however, and in support of the findings in this study, CD8+ T-cells are often the predominant T-cell immunophenotype in SS lesions [37], [38], [78]. Our prior demonstration that CD4+ and CD8+ T-cells in blister fluid elicited over SS lesions are predominantly of the memory and memory effector immunophenotype, expressing the activation marker CD38 [43], can be interpreted as an indication that populations of T-cell subsets in the skin of patients are antigen-specific. While naïve CD4+ T-cells are primed in the lymph-nodes by treponemal antigens via MHC-class II pathways [82], CD8+ T-cells will require some form of cross-presentation of spirochetal peptides via MHC-class I molecules [82], [83]. One plausible explanation for how cross-presentation might occur is that treponemal constituents enter alternative endocytic pathways in DCs or macrophages, allowing bacterial peptides to bind to MHC-class I molecules in the endoplasmic reticulum [82]. It is interesting to note, in this regard, that Bouis *et al*. [84] showed that DCs ingest virulent treponemes by coiling phagocytosis, an uptake mechanism that has been associated with cross-presentation [85]. An additional reason for this phenomenon is that circulating spirochetes could be internalized and cross-presented directly by lymph node-resident DCs; a highly specialized DC population which in humans can cross-present antigen without activation [86]. Because NK-cells promote the development of adaptive immunity via a bi-directional cross-talk between naïve CD4+ T-cells and DCs [42], [53], one could envision a model where alterations in circulating NK-cell populations, as shown herein, could interfere with adequate antigen presentation to CD4+ T-cells in the lymph nodes. Cross-priming of naïve CD8+ T-cells in SS could, thus, serve as a compensatory mechanism for less than optimal CD4+ T-cell priming in the lymph nodes of SS patients.

How CD8+ T cells are activated in the skin is not entirely clear since this subset typically responds to intracellular bacterial pathogens [87], [88]. Perhaps CD8+ T-cells are required to eliminate intracellular reservoirs of the bacterium that may be present in non-phagocytic cells from early syphilis lesions [89]–[91]. Then again, treponemal antigens could be cross-presented to cytolytic T-cells in the skin by tissue based macrophages and/or DCs inducing their activation. Given that cytokine producing CD56+ NK-cells were also enriched in the skin of patients, it is also plausible that this innate immune lymphocyte provides an additional source of IFN-γ in *Tp*-infected tissues. In support of this idea, we previously demonstrated that in Tp-stimulated PBMCs, NK-cells are a major source of IFN-γ [53] and showed that production of this cytokine is dependent on the presence of accessory cells (i.e., DCs). Lastly, in agreement with a recent report that IFN-γ and IL-17 producing CD8+ T-cells are present in the skin of SS patients [38], the transcript for IL-17 also was up-regulated in SS biopsies [38]. It is conceivable that IL-17 producing T-cells play an important compensatory role in SS, particularly in HIV-syphilis co-infected patients with very low CD4+ T-cell counts.

Although type I IFNs have generally been associated with antiviral immune responses, there is now compelling evidence that these cytokines also are induced in response to several intracellular [68], [92]–[95] and extracellular bacteria [23], [96], [97]. It was, therefore, not at all surprising that IFN-β and several type I IFN associated transcripts (**Table 3**) were up regulated in SS lesions. A variety of ligands, including bacterial DNA and RNA, can activate PRRs present in either the cell cytosol or membrane bound TLRs [68] to induce type I IFNs. In this regard, we recently provided evidence that transcription of IFN-β in human monocytes stimulated *ex vivo* with *Borrelia burgdorferi*, the Lyme disease spirochete, was dependent on phagocytosis and degradation of the bacterium and required signaling through TLR8 [23]. In support of a similar role for phagosomal signaling in *Tp*-mediated induction of type I INFs, three endosomal TLRs (TLR7, 8 and 9), capable of sensing nucleic acids within phagosomes of macrophages and DCs [98]–[100], also were markedly up-regulated in syphilis lesions. Type I IFNs are likely to have several important roles in the immune response to the spirochete. Firstly, type I IFNs can induce the differentiation of plasmacytoid DCs into mature antigen presenting DCs through their ability to up-regulate surface expression of co-stimulatory molecules like CD80, CD86, and CD40 [68]. Type I IFNs could also help regulate NK-cell function by inducing the production of IL-15 by macrophages, a cytokine which was increased in SS lesions and can promote NK-cell survival and proliferation [101]. Lastly, type I IFNs may facilitate cross-presentation of antigens via MHC class I molecules to CD8+ T-cells [65].

Based upon the findings from this and prior studies [43], we now propose a revised early syphilis pathogenesis model that integrates innate and adaptive immune responses to the bacterium and also takes into account the spirochete's immunoevasive countermeasures against host defenses. According to this model, spirochetes replicate at the site of initial inoculation unchecked by the innate immune surveillance system and rapidly disseminate to the skin and other tissues. At some point after initial entry of the bacterium increasing local spirochetal burdens allow a small number of organisms to be taken up by resident phagocytes, although this process is inefficient in the absence of opsonic antibodies. APCs containing phagocytosed spirochetes can then migrate into draining lymph nodes where they present treponemal antigens to naïve CD4+ T cells and B-cells. We postulate that neo-sensitized T-helper cells traffic back into the primary lesion, where they recognize their cognate antigens and release IFN-γ. Clearance of organisms by IFN-γ activated tissue macrophages is markedly facilitated by the emergence of high titers of *Tp*-specific opsonic antibodies. In parallel events, while the chancre resolves, as soon as treponemal loads in the skin of early syphilis patients reach a sufficient density capable of triggering the local inflammatory response, SS skin lesions become clinically apparent. In contrast to the immunologic events that initially take place in the primary chancres, innate and adaptive immune responses in SS skin lesions appear to co-evolve in the presence of both memory and memory effector CD4+ and CD8+ T cells and high titers of opsonic antibodies. One would thus predict that these changes would be sufficient for the immune response to eradicate the bacterium. However, the paucity of OMP antigenic targets on the outer leaflet of the bacterium together with the emergence of *Tp*-subpopulations resistant to opsonophagocytosis, permits varying numbers of bacteria to avoid opsonization, uptake and clearance by dermal macrophages. The low-level bacteremia which ensues allows the spirochete to avoid recognition by host innate and adaptive immune defenses in the blood compartment. The constant spread of *Tp* into other tissues during SS, specifically the bone marrow, could affect the development of myeloid and lymphoid progenitors of monocytes/macrophages, DCs and NK-cells. Fortunately for the host, over time, the emergence of greater numbers of activated memory and memory effector CD4+ and CD8+ T-cells, IFN-γ producing CD56+ NK-cells together with increasing titers of *Tp*-specific opsonic antibodies, allows the host to ultimately gain the upper hand against the bacterium. The complex shifting balance between immune evasion and bacterial persistence to immune recognition and spirochetal clearance, will thus, not only determine the intensity and duration of the clinical manifestations of venereal syphilis but also how long the spirochete can endure in blood and tissues.

## Supporting Information

Table S1 ***Tp*** **loads and IHC quantitation results.**
(XLSX)

Table S2 **Complete human inflammatory immune array results.**
(XLSX)

Table S3 **Complete human phagocytosis immune array results.**
(XLSX)
